# Psychoneurological Disturbances Affecting the Limbs

**Published:** 1918-08

**Authors:** G. Roussy


**Psycho-Neurological Disturbances Affecting the Limbs Observed During the War.** Professor G. Roussy, Médecin-Major de 2e classe, Médecin Chef du Centre Neurologique de la 7e Région, Professeur agrégé à la Faculté de Médecine de Paris. The speaker said that among the multitudinous manifestations of a psychoneurological description that the war has revealed, there is a group of disturbances which at the present time gives rise to divergence of opinion of great practical importance, i. e., the contractures or paralyses of the limbs, principally those limited to one segment of a limb and particularly to the extremities (acro-contractures or acro-paralyses).

For more than a year, in collaboration with Dr. Boisseau and Dr. d'Oelsnitz, he has given special attention to the study of these disturbances, and it is the result of their combined work that forms the subject of the present report. Some of it has already been published in various forms, but the remainder is new and will shortly appear in a book which is now in the press[1].

He first discussed the principal clinical types of contracture or of psycho-neurological paralysis which were commonly observed by French neurologists, and then described the nature and pathogenesis of a certain number of these nervous disturbances which have revealed themselves in the course of psychotherapeutical treatment

Clinical types. Psychomotor disturbances affecting a limb may be localized in its distal extremity, its middle third, or its proximal end.

I. *Psychomotor disturbances localized in the distal extremity of a limb. Acroparalyses and acrocontractures.*

1. Absolute paralysis of the hand or foot.
2. Local contractures.

In the case of the hand, the clinical picture comprises two symptomatic elements : 1. A motor element, contracture, paralysis or a combination of the two, with unlimited possibilities of variation, natural or unnatural; 2. Secondary phenomena which, clinically, may be divided into :

*a*) Lesions of bones and joints and of muscles and tendons. These are rare.

*b*) Caloric, vasomotor, secretory, trophic, and electric distur-

1. G. Roussy, Boisseau et M. d'Oelsnitz. *Traitement des psycho-névroses de guerre* Coll. Horizon, Masson, édit., 1918. (En préparation.)

bances. These are sometimes absent but are more usually found, either in combination or separately.

In the case of the foot, the condition is one of talipes varus, which, according to the character or predominance of the motor disturbance (contracture or paralysis) one may divide into three categories : 1. Paralytic talipes varus or foot-drop; 2. Reducible talipes varus; 3. Irreducible or blocked talipes, which is an accentuation of category 2.

II. *Psychomotor disturbances localized in the middle third of the limb. Elbow and knee.*

A. *Contractures limited to the elbow or to the knee alone.* These are clinically of two types : 1. the extended type and 2. the flexed type.

1. The extended type is rarely seen as affecting the elbow; in the knee it is not so frequently seen as the flexed type. That it is purely hysterical in origin can be proved by examination under anesthesia and by its amenability to treatment. There is, however, a diversion of this type resulting from an organic lesion and accompanied by secondary hysterical disturbances which can easily be distinguished from the purely pithiatic form by careful examination.

2. The flexed type has very frequently been observed during this war. It may appear under the most diverse forms, and it is for this reason that so many different interpretations have been put upon this class of vicious attitudes. The following is a useful grouping :—

*a*) Purely functional contractures. These are the least common, and are usually seen only in comparatively recent cases. They are the result of paradoxical wounds more often affecting the foot, the leg, or the thigh than the knee; and more often the shoulder, the forearm, or the hand than the elbow. They do not, as a rule, affect the articulation, and are generally of a hystero-traumatic origin. The clinical signs are limitation of movement, both extensor and flexor, with contracture or paradoxical contraction of the antagonistic muscles when spontaneous or voluntary movements are attempted. Secondary disturbances are common, particularly in cases of long standing. The class is one of vicious attitudes completely reducible by general anesthesia and by psychotherapy.

*b*) Primary functional contracture with secondary organic troubles of the articulations. This is the type most frequently seen at the present moment in the neurological centres of the interior. The vicious attitude is of the same etiology as the preceding type, and is produced by hystero-traumatism not necessarily directly affecting the elbow or knee joint. Clinically the condition is difficult and often at first impossible to diagnose. The situation may be summed up as follows :— The vicious position is partially reducible under anesthesia and by means of psycho-therapeutical treatment, and may be progressively reduced by psycho-physiological treatment.

*c*) Secondary functional contracture (or contraction), with primary articular lesion. From the pathogenic point of view this type is the inverse of the preceding one. Here there is a traumatic lesion or a disease of the elbow or knee which may be traced to its origin. The patient has originally been a " joint case " and has retained a vicious attitude of an antalgic character or one resulting from immobilization.

These various types must, of course, be differentiated from those in which the vicious attitude is totally irreducible under anesthesia and by psychotherapy, that is, cases of purely articular or purely musculo-tendinous lesions.

B. *Contractures of the elbow and knee combined with motor disturbances of other segments of the same limb.* Contracture of the elbow or of the knee may be associated either with a vicious attitude of the distal end (hand or foot) of the same limb, or with a contracture of the proximal end (shoulder or hip).

*a*) Sling arm. This type is shown clinically by a contracture of the elbow combined with flexion of the hand on the forearm as in pseudo-radial paralysis. It is a form frequently observed in patients whose arms have been too long maintained in slings, and is brought about by psychical fixation of such a position.

*b*) Crutch leg (flexed knee and talipes equinus).

In both these types examination under anesthesia enables the part played by organic or functional disturbances respectively to be estimated.

III. *Psychomotor disturbances localized in the roots of the limbs, shoulder, and elbow.*

As in the case of the elbow and knee, all flaccid forms which come into the category of brachial or crural monoplegia must be eliminated and only forms in which contractures are present considered.

In the shoulder two forms are seen :

(1) Adduction type, in which the arm is held more or less rigidly to the body, though the elbow, wrist, and fingers move normally. Fairly frequently there is also elevation of the shoulders by contracture of the elevator muscles, a type which is associated with the following, or

(2) Elevation type. This is rarer than the preceding one, especially in its pure, isolated form. Contracture of the trapezius and scalenus fixes the shoulder in forced elevation. Contraction of the sterno-mastoidal often becomes an additional feature in this condition and results in a species of torticolis.

In the hip the following three forms occur :

(1) External rotation and abduction type. This is the most common. There is of course no real shortening of the affected limb.

(2) Flexion type.

(3) Pseudo spring-hip type. This is not so much a nervous form as an anatomical arrangement which allows the subjet to tighten the tendon of the fascia lata on the great trochanter at each step, and to produce or suppress the spring effect at will. As a matter of fact the condition is the first stage of true spring-hip, in which the looseness of the tendons cannot be voluntarily corrected.

As in the case of the knee and elbow, one finds distinct forms according to whether the organic or functional element is predominant.

*a*) Purely functional contractures. These may be reduced under anesthesia, and recover completely by psychotherapy in the great majority of cases.

*b*) Primary functional contractures with secondary articular lesions. These are more frequent in the shoulder than the hip. As they are difficult to distinguish from the preceding type, recourse must be had to examination under chloroform. In the same degree as they are only partially reducible under anesthesia according to the established stiffness of the joint, so they are but partially amenable to psychotherapy and only give way progressively to psycho-physiological treatment.

*c*) Functional contractures (or contractions) secondary to primary lesions of the joints. A purely psycho-neurological element is here superimposed upon a traumatic or spontaneous articular lesion as a result of too long immobilization, habit, or psychic inertia.

Lastly, the contractures we are studying are :

(1) Sometimes limited to the hip or to the shoulder, the vicious attitudes produced in other segments of the limb being temporary and vicarious;

(2) Sometimes, on the other hand, such vicarious attitudes are themselves fixed as the result of later and additional functional disturbances, in the form of limb paralysis or of contracture.

As regards the nature of the motor disturbance, it is pithiatic and may be reproduced or maintained at will by the patient. It is liable to disappear suddenly by persuasion.

In our experience, these objections are either unjustified in practice or originate in factors which oppose, in one way or another, the treatment applied.

Among the disturbances classed as reflex are *sensory disturbances,* or anesthesia and hyperesthesia. In our opinion, however severe these affections may be, they are all hysterical in character. In some cases they may be real in that they are due to a stiffness of an articulation, particularly in the phalanges; but when treatment has improved the condition of the joint, the sensory trouble disappears.

*Vasomotor, thermic, and secretory disturbances* are frequently, but not always, observed in connection with paralysed or contracted extremities. They appear to be caused by the immobilization or the vicious attitude acquired by a limb or segment of a limb, and in proof of this theory we would urge the fact that on the return of the physiological mobility these troubles either disappear completely or are considerably improved.

*Mechanical or electrical hypersensitiveness of the muscles and nerves and modifications of the cutaneous reflexes* is believed to be due to hypothermia and disturbances of the circulation. They are increased by cold, and become accentuated or even disappear under the influence of heat. They also disappear as soon as removal of the motor trouble has allowed the circulation of the limb to return to normal.

*Trophic disturbances of the skin, muscles, and bones, and of articular lesions* are still of obscure origin and admit of many hypotheses. Here also, in his opinion, sufficient importance has not been given to the role played by immobilization in the abnormal conditions it imposes upon the vascular and thermic regulation systems of a limb deprived for months or even years of any kind of movement, and during which period it is sheltered both from light and air. The skin, nails, and hair undergo modification as soon as movement is recovered, and this factor alone proves to how great an extent immobilization must be considered responsible for other changes.

*Mental condition.* It seems to us that sufficient stress has not been laid in recent years on the great influence exerted by the mental condition in the genesis of psychoneurological affections. The soil in which hysterical disturbances have been cultivated should be carefully studied with a view to comprehension of the psychical state of the patient, his *moral,* his personal disposition, as we consider that in a satisfactory mental condition lies the primary element of recovery from psychoneurological disturbances.

Reflex disturbances may be considered as phases of hysteria associated with secondary phenomena.

They develop in subjects giving evidence of:

A special psychic condition, previously existing or more often acquired or accentuated by the circumstances under which they are living. This psychic condition, which ranges from the mental state of the pithiatics to that of the victims of factory accidents and even to that of the claimants of legal rights (Brissaud's " sinistroses " or sufferers from misfortune), gives rise, on the occurrence of traumatism or some other factor, however slight or commonplace, to the appearance of motor disturbance of a hysterical nature; this may be voluntarily repeated and is equally liable to disappear suddenly if persuasion is brought to bear. Should such an influence be exerted on a limb whose circulation is perfectly normal, without incurable direct muscular lesions, this motor trouble may be the only symptom; it is a purely pithiatic disturbance. If, however, it develops in a previously existing abnormal circulatory field, acquired or accentuated by military life or by any other cause (arterial lesions due to the garrot, for instance) it may give rise to secondary phenomena. This defective circulation, doubtless connected with a disorganization of the sympathetic nervous system, consists in a vaso-constriction which is generally revealed by a bilateral diminution of the range of the arterial oscillations (microsphygmically).

On the ground thus prepared, immobilization or vicious habits in the use of the limb, due to the motor disturbance, then come into play, exaggerating the microsphygmia on the affected side (" amicroanisosphygmia ").

The latter gives rise to cyanosis and disturbances of the thermal system and their consequences, viz. deterioration in the mechanical and electrical contractility of the muscles, secretory and trophic changes, and alteration in the cutaneous reflexes.

Other factors (especially immobilization and the after-effects of infection) then begin to work on the ill-nourished limb, and create the lesions of the muscles and tendons which result in retractions, and the lesions of the bones and joints which cause fibrinous ankylosis.

Inactivity, insufficient nutrition, and sometimes a reflex reaction may bring about a muscular atrophy of the limb which, in its turn, affects the reflexes.

*Conclusions and practical deductions.*

Though we have considered it necessary to go very fully into the question of the pathological origin of reflex disturbances it has not been solely in response to the natural tendency to expose the results of our personal observations here; it is rather because a very useful practical lesson may be derived therefrom.

Whether or not one of the pathogenic theories described above be adopted (though such theories form only a part of the hypotheses which may be confirmed or disproved), an initial idea of considerable importance appears to issue more clearly before the Neurological Society than during the last two years. At first regarded doubtfully, and even rejected altogether by many doctors, the idea of Messrs. Babinski and Froment as to the possibility of successfully treating the physio-pathological syndrome through its chief element — the motor disturbance — is now accepted with almost complete unanimity.

Whether the reflex action, by persisting for a greater or lesser time after the disappearance of the original injury, exaggerate the vicious position of a limb or part of a limb into a contracture or paralysis (the theory of Babinski and Froment); whether the reflex action be only temporary and a psychic mechanism of fixation soon be substituted for it (the theory of Claude and Lhermitte); or whether the vicious attitude (paralysis or contracture) be from the beginning only the effect of a defensive movement or contraction in the presence of trauma, favored by emotion and fixed by suggestion, like all hystero-traumatic affections (the theory of Roussy, Boisseau and d'Oelsnitz), the psychic element dominates the clinical picture sooner or later. Whether this element be frequent and always secondary (the hysterical reflex association of Babinski and Froment); whether it be consistent and primary (as is thought by Roussy, Boisseau, d'Oelsnitz, Claude and Lhermitte), there is no doubt as to the therapeutic procedures to be instituted and as to the necessity for all medical men to have recourse to psychotherapy.

The idea of curability merits a wide expansion, not only among neurologists, specialists and other doctors, but also among our wounded, who are, unfortunately, too much inclined to exaggerate the seriousness of their disability with an ulterior object.

It is towards this end that our personal efforts have been directed for more than a year.

Over and above the possibility of curing physiopathological affections, another and not less important idea becomes inevitable: it is admitted (as we already admit) that the physical disturbances, slight or serious (cyanosis, hypothermia, stiffness, and ankylosis, tendon retractions, amyotrophia), are only secondary phenomena directly due to the motor trouble, and that they appear, in the great majority of cases, only at a very late stage. This idea is that more often than not such affections are avoidable if treated early enough, and if the contracture or paralysis is taken in hand as soon as it gives evidence of a tendency towards fixation.

Finally, we may say that real hysterical disturbances or hysterical disturbances associated with secondary lesions of an anatomical character, both belong to the classical school of traumatic hysteria and call for the same therapeutical treatment (psychotherapy or psycho-physiotherapy) and the same prophylactic measures.

